# Progress in Neurology

**Published:** 1900-12-15

**Authors:** 


### Progress in Neurology.

**Influenza and the Nervous System.**—In introducing a discussion on this subject at the annual meeting of the British Medical Association, Bury[1] said that the nervous system is affected in some degree in nearly every well-marked case of illness due to the influenza bacillus. Thus, during the febrile stage there are usually severe pains in the head, back, and limbs, and the muscles are tender even to slight pressure. There is often restlessness and inability to sleep, and there may be delirium. The subjective feelings of intense misery and depression are also in all probability the result of influenza toxins acting on nerve tissue.

In some cases, however, the nervous system seems to be specially selected for attack by the bacilli and their poisonous products. Such cases may be broadly separated into two groups: in the first may be placed those in which nervous diseases develop during or shortly after the febrile stage—meningitis and hæmorrhagic encephalitis are the best examples. In the second group we may place nervous diseases which usually occur after the attack has subsided—neurasthenia and multiple neuritis are good instances. Such a division, though not strictly accurate, is clinically convenient.

Of the nervous symptoms belonging to the first group the most important are those which indicate a morbid condition of the brain. Two types can be distinguished—the comatose and the delirious. In the comatose, a patient, with or without the usual symptoms marking the onset of influenza, *i.e.* prostration, pyrexia, headache, nasal catarrh, gradually becomes drowsy and apathetic, and in a few days becomes comatose. Recovery may occur, though a fatal termination is more common. Examination of the brain in such cases may reveal nothing abnormal, or a purulent meningitis with or without encephalitis, which is usually hæmorrhagic in character. In contrast to the drowsiness, somnolence, and unconsciousness which characterise the comatose type, we may meet with cases in which restlessness, irritability, delirium, and even mania are the essential features. The prognosis is better than in the comatose type. In infants an attack of influenza may be represented solely by pyrexia and convulsions.

The nervous sequelæ of influenza are numerous—almost every possible variety of functional, inflammatory, and degenerative disease of the nervous system may occur. Thus we may have neurasthenia, hysteria, epilepsy, outbursts of delirium, and every variety of psychosis, meningitis, encephalitis, cerebral hæmorrhage, and thrombosis. In the cord almost every variety of mychtis may occur. And there may be neuralgia or neuritis of almost every nerve.

In discussing this paper Broadbent said that some of the nervous sequelæ, *e.g.* insanity, of influenza seem to be attributable rather to antecedent instability of the nervous system than to anything specific in the action of the influenza poison, as they may also follow typhoid and other acute diseases. Allbutt said that the diagnosis of influenza in these cases was often doubtful, but a clinical feature which may perhaps be called decisive is the suddenness of onset. In regard to prognosis of influenza of the nervous system, strong assurances of recovery cannot be given, for though the large majority recover, in a substantial remnant of cases the nervous parts affected are permanently damaged. Newton Pitt said that many of the cases of meningitis attributed to influenza are not directly due to it; but after an attack of influenza patients are much more liable to take certain diseases to which previously they were immune: such as enterica, and probably in this way they more readily fall victims to the diplococci of Weichselbaum and of Fraenkel, either of which may be the efficient cause of the meningitis. The great cardiac irregularity and failure not infrequently met with after influenza may be due to affection of the cardiac ganglia and nerves quite as much as to a lesion of the muscle. Not infrequently, however, the cardiac disturbance and profound depression which are met with are due to a marked dilatation of the stomach with decomposition of its contents. Buzzard, in illustration of Broadbent's statement, mentioned a case of tabes of some years' duration where the patient, who had so much improved as to find no difficulty at all in the use of his legs, was suddenly rendered completely atoxic by an attack of influenza. Yet such was by no means commonly the case, and the diseases in question were in all probability the effects of the toxin of influenza on previously healthy structures. This could be supported by analogy; in influenza, just as in syphilis of the nervous system, the symptom-complex was often bizarre—there might be, say, paralysis of an oculo-motor nerve alongside of paraplegia. This peculiar character strongly suggested the action of a toxin on the nervous system. He thought that the number of grave diseases attributable to toxæmia had greatly increased during the past ten years, and a large proportion of these were probably due to influenza.

**Cerebro-spinal Meningitis.**—During the latter part of the year 1899 and the spring of 1900 an epidemic of cerebro-spinal meningitis occurred in Dublin. At a meeting of the Royal Academy of Medicine in May a good many of the cases were recorded and discussed. Drury, who had had eight cases, said that the present epidemic was of a comparatively mild type when compared with the Dublin one of 1866–67, which was exceedingly fatal and very often attended with a hæmorrhagic rash.

The symptoms found in the present epidemic were, briefly, those of meningitis with some special peculiarities. Of these early retraction of the head was important and very constantly seen; the spasm might extend to the back muscles and cause opisthotonos. These conditions were produced by irritation of the spinal nerves as they passed through the membranes. To the same cause was due the pain complained of, especially in the limbs, and the hyperæsthesia of the whole surface of the body. Herpes was very frequently present and often extensive; it might appear not only on the lips and nose, but on the other parts of the body. Inflammation of the conjunctivæ, of the iris, or of the whole ureal tract leading to destruction of the affected eye was not unfrequently seen. Deafness was also frequent, with or without purulent discharge from the ear. Recovery from this might occur wholly, in part, or not at all. Not unfrequently there was swelling and redness of some of the joints, like that of acute rheumatism. This might be due to a serous effusion, or to a purulent one, which might disorganise the joint. It was not uncommon to meet with either broncho- or lobar-pneumonia. By bacteriological examination it was proved that these were not merely cases of ordinary pneumonia with accidental meningitis, but that the pneumonia was a complication of the meningitis.

Bacteriological examination of fluid drawn off by lumbar puncture showed the presence of the diplococcus intracellularis meningitidis.

Parsons and Littledale recorded seven cases, of whom three died. They remarked that the onset was sudden and definite; as a rule the patient can fix to the hour the time that he was taken with the headache. There was no constant type of temperature; in some it resembled typhoid, in others malaria. The patient generally lay on his side with his head retracted, chin pointed, and lower extremities flexed on the abdomen. Kernig's sign was invariably present. The knee jerk was diminished or absent, never exaggerated. Four of the seven had well-marked nasolabial herpes. Most of the cases had strabismus and double vision, always due to the external rectus being involved if recovery took place control over the rectus was regained. There was always some involvement of the fundus. Ice and evaporating lotions were applied to the head. Trional, bromides, and morphia were used to relieve restlessness and procure sleep. A camphor and chloroform liniment rubbed in seemed to greatly relieve the pain.

Moore, at the Meath Hospital, had had eleven cases, with three deaths. He said that in the cases that recovered the emaciation was very great. The present epidemic was remarkable for the decubitus, the most marked opisthotonos being present. As to treatment he had found that the most successful measures were leeching behind the ears, and the repeated application of small blisters behind the ears. The agonising headache was relieved by a combination of acetanelide, caffein, and sodium bicarbonate.

Travers Smith had had fourteen cases, of whom four died. He had also found the sudden onset, retraction of the head, irregular temperature recorded by previous speakers. Eight of the fourteen cases had eruptions early: those most common were an erythematous eruption and herpes. The erythematous eruption was bright red, with well-defined edges, felt very hot, and persisted for a good many days. It was not uncommon on the neck, but in one case it appeared on the ankle, and in another on the breast and down the arms to the elbow. The herpes was most common on the lips and face. In only one case was there an approach to the classical description of purpuric spots The hydrocephalic cry was only found in children Emaciation was very rapid; despite the fact that they got more food, they wasted more rapidly than typhoid patients. There was a tendency to intermissions and exacerbations. In the cases which showed this, a rise in temperature was succeeded by headache and vomiting. The duration was variable—one case not yet quite well had lasted seventy-even days, one proved fatal on the eightieth day. One patient died within a day and a half. The only sequela was permanent deafness in one case. Prognosis was very difficult, some of the apparently worst cases recovered, while, on the other hand, two had suddenly become comatose and died within an hour, one on the seventeenth, the other on the second day.

Handford,[3] of Nottingham, has described five cases of sporadic cerebro-spinal meningitis occurring in that city. He had also heard of five others. The ages of the patients were three, four, five, fourteen, and twenty-two years. One proved fatal after fourteen weeks' illness, the others recovered; one was complicated by suppurative choroiditis. The cases formed a striking group, with many marked points of resemblance. The most prominent symptoms common to all were:—(1) Rapid, if not actually sudden onset, without injury or previous failure of health. (2) Fever: the temperature rose at once to 102°–104° and very slowly subsided, sometimes showing distinct and regular intermissions. In three cases (one fatal) the temperature remained normal for weeks, without any other sign of improvement. (3) The intensity of pain in the head and neck, aggravated by the slightest movement. (4) Marked retraction of the head. (5) Muscular hypertonicity, twitching, and rigidity. (6) Extreme and rapid emaciation. (7) Tendency to relapse and to intermissions.

The cases differed in their clinical course very markedly from tubercular meningitis. The onset was more acute, the retraction of the head more marked, the duration of the disease much longer, and the percentage of recoveries far greater. None of the five cases had herpes, and only one showed some patchy erythema. Clinically the distinction between individual cases of posterior basic meningitis, sporadic cerebro-spinal meningitis, and epidemic cerebro-spinal meningitis is impossible. Bacteriologically, the distinctions between the diplococcus intracellularis meningitidis, found in the first-named disease, and in the other two seems chiefly one of degree.

As regards treatment morphine was given in full doses. In three cases potassium iodide appeared to do good; in one it was evidently prejudicial, and in one without distinct effect. Morphine appeared to do good beyond the mere relief of pain.

[1] Brit. Med. Jour., Sept. 29, 1900. [2] Dub. Med. Jour., July, 1900. [3] Brit. Med. Jour., July 14, 1900.

